# IMPACT OF ATOPIC DERMATITIS ON THE QUALITY OF LIFE OF PEDIATRIC PATIENTS AND THEIR GUARDIANS

**DOI:** 10.1590/1984-0462/;2017;35;1;00006

**Published:** 2017-02-20

**Authors:** Amanda Letícia Bezerra Campos, Filipe Moreira de Araújo, Maria Amélia Lopes dos Santos, Alex de Assis Santos dos Santos, Carla Andréa Avelar Pires

**Affiliations:** a Universidade do Estado do Pará (UEPA), Belém, PA, Brasil.; b Instituto Bioestatístico, Belém, PA, Brasil.

**Keywords:** Dermatitis, atopic, Quality of life, Pediatrics

## Abstract

**Objective:**

To evaluate the impact of atopic dermatitis on the quality of life of pediatric patients in the age group of 5-16 years, and their parents, assisted at the Dermatology Department of *Universidade do Estado do Pará* in 2015.

**Methods:**

A cross-sectional study including 51 patients and their guardians, to whom two questionnaires about the quality of life were applied, the Children’s Dermatology Life Quality Index (CDLQI) and the Dermatitis Family Impact (DFI). To evaluate the severity of the disease, the researchers applied the Severity Scoring of Atopic Dermatitis (SCORAD) index. The Pearson Product-Moment Correlation Coefficient (PPMCC) evaluated the correlation between CDLQI, DFI, SCORAD, and the contingency coefficient C evaluated the association between the qualitative variables, considering *p*<0.05 significant.

**Results:**

Of the patients, 55% were female. The average age was 9.5±3.2 years, and 41% had family income up ≤1 minimum wage. The average score was 5.4±5.1 for CDLQI, 6.6±4.5 for DFI, and 28.3±19.8 for SCORAD. The correlation among the scores CDLQI, DFI, and SCORAD was significant by the PPMCC (*p*<0,001).

**Conclusions:**

Atopic dermatitis affects the quality of life of both children and their guardians, and indicates the importance of including the study of quality of life as a complement to clinical evaluation.

## RESUMO

**Objetivo:**

Avaliar o impacto da dermatite atópica na qualidade de vida de pacientes pediátricos de 5 a 16 anos e seus responsáveis, atendidos no serviço de dermatologia da Universidade do Estado do Pará (UEPA) em 2015.

**Métodos:**

Estudo transversal de 51 pacientes juntamente com seus responsáveis, aos quais foram aplicados dois questionários de qualidade de vida, o Escore da Qualidade de Vida na Dermatologia Infantil (CDLQI) e o Impacto da Dermatite Atópica na Família (DFI). Para avaliar a gravidade da doença, os pesquisadores aplicaram o índice de *Severity Scoring of Atopic Dermatitis* (SCORAD). A correlação linear de Pearson foi aplicada para averiguar a correspondência entre os instrumentos CDLQI, DFI e SCORAD, e o coeficiente de contingência C para avaliar a associação entre as variáveis qualitativas. Considerou-se significante *p*<0,05.

**Resultados:**

Dos pacientes, 55% pertenciam ao sexo feminino. A idade média foi de 9.5±3.2 anos, e 41% tinha renda familiar de até um salário-mínimo. A média dos escores foi de 5.4±5.1 para o CDLQI, 6.6±4.5 para o DFI e 28.3±19.8 para o SCORAD. Atestou-se correlação altamente significante entre os escores CDLQI, DFI e SCORAD pela correlação linear de Pearson (*p*<0.001).

**Conclusões:**

A dermatite atópica afeta a qualidade de vida tanto das crianças quanto de seus responsáveis, o que indica a importância de inserir o estudo da qualidade de vida de forma complementar à avaliação clínica dos pacientes.

## INTRODUCTION

Atopic dermatitis (AD) is a chronic inflammatory dermatosis of multifactorial etiology, characterized by moderate-to-intense pruritus. This condition evolves to outbreaks, and has a hereditary allergic character.^1^

In the past three decades, the number of patients with AD has doubled - or even tripled - in most parts of the world, constituting a major public health problem, especially in industrialized countries.^2^ ^,^ ^3^

In Brazil, the prevalence of AD ranges according to the affected age group and Brazilian region analyzed. The North and the Northeast have a slight higher number of cases, and in all places, the prevalence is higher among younger children (6 and 7 years old). The International Study of Asthma and Allergy Diseases in Childhood (ISAAC) was conducted in Brazil and pointed to mean prevalence of AD of 7.3%, expressed as severe in 0.8% of the patients in the age group from 6 to 7 years. At the age of 13 and 14 years, the mean prevalence of AD was 5.3%, and severe AD, 0.9.^4^

The diagnosis of AD is essentially clinical and is based on clinical-laboratorial diagnostic criteria established by Hanifin and Rajka.^5^ Complementary tests can help, but are not sufficient to define a diagnosis.^6^

Skin conditions have a negative impact on emotional status, on social relationships and on daily activities, thanks to the stigma caused by the appearance of the lesions.^7^ Chronic pruritus is often untreatable, so, it has a major impact on the quality of life of the patient, since it is negative for the quality of sleep, affecting children’s behavior by day as well as their productivity.^8^ There is also the social, emotional, and financial impact on the patients’ families. Parents of the affected children report difficulties in discipline and care of their children, generating conflict between parents and healthy children, thus changing the family structure.^9^ ^,^ ^10^

In this context, this study aimed at measuring, through validated questionnaires, the impact of AD on the quality of life of pediatric patients in the age group of 5-16 years and their guardians, assisted in a reference pediatric dermatology service located in Eastern Amazon.

## METHOD

This is a descriptive, cross-sectional study, including pediatric patients in the age group of 5-16 years, of both sexes, diagnosed with classic clinical criteria of AD^5^ and assisted at the pediatric dermatology service at Universidade do Estado do Pará (UEPA), from February to August 2015, and their guardians. This service sees about 120 children with AD per semester. Fifty-one children who attended an appointment during the period, and who accepted to participate in the study with their parents, were included in the investigation. Therefore, sampling error was 10.5%.

Further to the approval in UEPA’s Ethics Committee, in February 2015, report number 960,962, the study was performed with the consent of the institution and the patients and/or their guardians, through an informed consent form and an informed assent form for adolescents in the age group of 12 to 16 years.

The severity of the disease was analyzed using the Severity Scoring of Atopic Dermatitis Index (SCORAD). Its assessment is based on the affected surface, and is calculated by using the rule of 9, the same used for burns (one child with half of the arm affected has 4.5% of the body surface affected by lesions, since an arm corresponds to 9% of the total body surface, for example). It is also based on the intensity of the eczema, by the presentation of elementary lesions (erythema, edema/papule, exudate/wounds, excoriations, and lichenification), and on the repercussion of subjective symptoms of pruritus and sleep loss.^11^ One SCORAD below 20 indicates mild AD (a few inflammatory crises); between 20 and 40 is classified as moderate AD (intense inflammation and pruritus); and above 40, severe AD (extensive, inflammatory, and frequent crises).^11^

Two questionnaires were used to assess the quality of life: the Children’s Dermatology Life Quality Index (CDLQI) and the Dermatitis Family Impact (DFI) both English and validated in the United Kingdom.^12^ ^,^ ^13^

The CDLQI, validated to spoken Portuguese in Brazil,^14^ is composed of ten questions regarding different aspects of life affected by the disease in the past week, involving six domains: symptoms and feelings (questions 1 and 2); leisure (questions 4, 5 and 6); school or holidays (question 7);personal relationships(questions 3 and 8); sleep (question 9); and treatment (question 10). Each question is scored as follows: very much=3; quite a lot=2; only a little=1; not at all=0. Total score ranges from a maximum of 30 and a minimum of 0, with values of 0 and 1 indicating no effect on quality of life; 2 to 6, a small effect; 7 to 12, a moderate effect; 13 to 18, a very large effect; and 19 to 30, an extremely large effect. The questionnaire was filled out by the children, assisted by the researchers, who read the questions aloud when necessary and answered their doubts about the questions. Some participants answered the questions verbally, and their answers were written down by the interviewers. The guardians did not interfere in the responses. Originally, 54 children were assessed, but two of them were excluded for having difficulties in understanding and for not accepting to participate; another one was excluded for undergoing a simultaneous treatment for psoriasis, which led to 51 children involved in this study.

The DFI helps to measure how much family life is impacted by a child with AD. It is designed to be filled out by the children’s guardian and has ten questions, all referring to the week before and related to housework, preparation of food, preparation and feeding, sleep, family leisure activities, expenditure, tiredness, emotional distress, and relationships. It was translated and culturally adapted to Brazilian Portuguese.^15^ The score attributed to each question and the final score are similar to CDLQI. In both questionnaires, the higher the score, the worse the reflection of AD on the patient and his/her family.

The sample was characterized by descriptive and inferential statistical methods. Qualitative variables presented Gaussian distribution, by the methods of D’Agostino-Pearson. Pearson’s linear correlation tests were used, as well as the contingency coefficient C for the correspondence between instruments CDLQI, DFI, and SCORAD. Alpha error was established at 5% for the rejection of a null hypothesis. Statistical processing was performed with BioEstat 5.3.^16^

## RESULTS

In the sample comprising 51 patients, 55% were girls and 45% were boys, with mean age of 9.5±3.2 years, ranging from 5 to 16 years. By categorizing the sample in age groups, it was possible to observe that 51% was in the age group of 5-9 years old, and 49% were aged between 10 and 16 years. The most common family income was up to one minimum wage (41.2%), followed by those with income from two to five minimum wages (39.2%).

Instruments of data collection presented the following means and standard deviations: CDLQI=5.4±5.1; DFI=6.6±4.5; and SCORAD=28.3±19.8. By assessing the quality of life of the children, CDLQI scored mostly the following specific questions (mean±standard deviation): symptoms (1.06±0.41), feelings (0.69±0.45), sports (0.65±0.48), personal relationships (*bullying*) (0.59±0.47), sleep (0.59±0.45), and leisure activities (0.57±0.40). When classified by domains, the highest score was that of symptoms and feelings (0.87), followed by sleep (0.59) and leisure (0.55). Table 1 shows the distribution of patients as to the severity of AD and the answers obtained in the questionnaires CDLQI and DFI.

**Table 1:** Distribution of patients as to the severity of Atopic Dermatitis and the answers obtained in the questionnaires CDLQI, DFI and SCORAD.

Questionnaires	n	%	*p*-value
CDLQI			
No effect	12	23.5	<0.001^a^
Weak effect	23	45.1	
Moderate effect	11	21.6	
Strong effect	4	7.8	
Very strong effect	1	2.0	
Total	51	100.0	
DFI			
None	10	19.6	<0.001^a^
Weak effect	14	27.5	
Moderate effect	21	41.2	
Strong effect	5	9.8	
Very strong effect	1	2.0	
Total	51	100.0	
SCORAD			
Mild	20	39.2	0.327
Moderate	19	37.3	
Severe	12	23.5	
Total	51	100.0	

CDLQI: Children’s Dermatology Life Quality Index; ^a^chi-square of adherence for expected equality of proportions; DFI: dermatitis family impact; SCORAD: severity scoring of atopic dermatitis.

The correlation between the severity of the disease (SCORAD) and quality of life (CDLQI) was first analyzed according to the classification in qualitative variables (categorical), using the contingency coefficient C, which led to *p*<0.001 (highly significant). It leads to the tendency of simultaneous moderate SCORAD and weak CDLQI (25.5%). Complementarily, the analysis of numerical scores obtained in each instrument (CDLQI and SCORAD) was conducted by Pearson’s linear correlation, which resulted in *p*<0.001* (highly significant), certifying the existence of positive and moderate correlation (r=0.680) between the scores of the instruments CDLQI and SCORAD (Table 2).

**Table 2:** Correlation between the severity of the disease (SCORAD) and quality of life (CDLQI) of pediatric patients with atopic dermatitis assisted at the dermatology service of Universidade do Estado do Pará (UEPA), February to August 2015.

CDLQI	SCORAD			
	Mild n (%)	Moderate n (%)	Severe n (%)	General n (%)
No effect	11 (21.6)	1 (2.0)	0	12 (23.5)
Weak effect	8 (15.7)	13 (25.5)	2 (3.9)	23 (45.1)
Moderate effect	1 (2.0)	3 (5.9)	7 (13.7)	11 (21.6)
Strong effect	0	1 (2.0)	3 (5.9)	4 (7.8)
Very Strong effect	0	1 (2.0)	0	1 (2)
Total	20 (39.2)	19 (37.3)	12 (23.5)	51 (100.0)

SCORAD: Severity Scoring of Atopic Dermatitis; CDLQI: Children’s Dermatology Life Quality Index.

The evaluation of the adjustment between severity of the disease (SCORAD) and quality of life of the guardians (DFI), demonstrated as categorical variables, was shown by the contingency coefficient C, which resulted in *p*=0.078 (not significant). That is, it was not possible to find a qualitative classification chart of the conjoint analysis of SCORAD and DFI (Table 3). The analysis of numerical scores of the instruments DFI and SCORAD using the Pearson correlation showed a positive and moderate correlation (r=0.512; *p*<0.001*) between the scores of the instruments DFI and SCORAD.

**Table 3:** Correlation between the severity of the disease (SCORAD) and the quality of life of guardians (DFI) of pediatric patients with atopic dermatitis assisted at the dermatology service of Universidade do Estado do Pará (UEPA), February to August 2015.

DFI	SCORAD			
	Mild n (%)	Moderate n (%)	Severe n (%)	General n (%)
No effect	6 (11.8)	3 (5.9)	1 (2)	10 (19.6)
Weak effect	7 (13.7)	5 (9.8)	2 (3.9)	14 (27.5)
Moderat eeffect	7 (13.7)	9 (17.6)	5 (9.8)	21 (41.2)
Strong effect	0	1 (2)	4 (7.8)	5 (9.8)
Very strong effect	0	1 (2)	0	1 (2.0)
Total	20 (39.2)	19 (37.3)	12 (23.5)	51 (100.0)

SCORAD: Severity scoring of atopic dermatitis; DFI: dermatitis family impact.

The correlation between the quality of life of the patient (CDLQI) and the guardian (DFI) was assessed by the contingency coefficient C for categorical variables, obtaining significance (*p*-value=0.012*) for the simultaneous occurrence of weak effect on the quality of life of the patient and moderate on the quality of life of the guardian (19.6%). The analysis of numerical scores of each instrument (CDLQI and DFI) by the Pearson correlation showed the existence of positive and moderate correlation (r=0.619; *p*-value<0.001*) between the scores of both instruments.

The drugs mostly used by the patients were emollients (56.9%), followed by topical steroids (51%) and systemic anti-histaminic (21.6%). Among topical steroids, the most frequent was mometasone furoate, a potent corticosteroid.

## DISCUSSION

This study demonstrated the correspondence between the severity of the disease (SCORAD) and the quality of life of the patient (CDLQI) and the respective guardian (DFI).

Most children did not use any medication, which differed from the reports of another study, conducted with a sample of children living in Europe.^17^ In that study, only 10% was not undergoing a drug treatment, probably because of the severity of AD in the patients investigated in that case (prevalence of moderate and severe cases), when compared to the patients in this study (more mild and moderate cases).

The emollient was mostly used, since dry skin is very common in AD, besides being a diagnostic criterion. In this sense, emollients are part of the atopic treatment to fight xerosis, and can also have an effect on pruritus and pain.^18^

The article of CDLQI validation^14^ obtained a mean score similar to the one exposed here, and showed that the two first questions, related to the domain of symptoms and feelings, seem to be the most important ones for the final score, as reported in the original instrument. The same data were shown in this study, which also reached higher scores in this domain, thus ratifying its importance.

This study showed similar results in relation to the questionnaires applied in Korea, in a dermatological clinic affiliated to a university hospital with 197 children,^19^ especially concerning the items in CDLQI: symptoms, sleep, and feelings.

By classifying CDLQI in domains, another study - Brazilian - conducted in Porto Alegre (RS),^20^ obtained the following in the three first positions: domain of symptoms and feelings, sleep, and treatment. The two first domains with the highest score were the same presented in this study. However, the treatment had the last position in our study because of the expressive percentage of patients who were not undergoing any treatment at the time the questionnaire was applied.

A study carried out in a tertiary center obtaines a mean SCORAD index of 36 (standard deviation: 16.2),^21^ which was highr that the index obtained in our study (mean: 28.3; standard deviation: 19.8). This difference may be because this institution treats more severe cases, since patients with mild AD are usually cared for in primary centers, general pediatric clinics, or dermatological outpatient clinics, like in this case. Likewise, the same longitudinal investigation reached higher means, in the first and second interviews, respectively, of CDLQI (10±6.6; 7.6±6.2) and DFI (9.4±5.3; 7.8±4.8), when compared to this study.^21^

The correlation between DFI and CDLQI using the Pearson correlation coefficient was positive and moderate, in accordance with the Brazilian and the Swedish studies, which also pointed to a significant correlation between these scores.^22^ ^,^ ^17^ In a study conducted in Italy, there was a high and significant correlation between the quality of life of children in the age group of 1-12 years and their guardians.^23^ This shows that the higher the score of CDLQI, the higher the DFI. When presented as ordinal qualitative variables (none, weak, moderate, strong, and very strong), there was a tendency for the weak effect on the quality of life of the patient, and moderate effect on the quality of life of the guardian.

In this study, most children fit the category “weak effect” on the quality of life and most guardians suffered moderate to very high effect.^22^ The data reflect how the presence of a child with AD affects the quality of life, leading to a high level of compromise in the family. The condition may even have more influence on the family dynamics than on the quality of life of the child, alone. This lower effect on the quality of life of the children in relation to the guardian may be due to the fact that the children can let go off easily, the difficulties of the disease.

The education of all individuals involved in children care is essential to handle AD. It is important to provide simple and clear information, without ambiguities, with the objective of reducing the negative impact on the quality of life of the family, since the lack of information about the disease and its treatment increases parental anxiety and makes it more difficult to adhere to the treatment and to general care, essential for therapeutic success.^23^

Another study using SCORAD and DFI as tools demonstrates that the quality of life of the family is correlated with the severity of AD in the child in an inversely proportional manner. That is, the higher the SCORAD, the lower the quality of life of the family, as shown in this study.^24^

An investigation about AD in the family environment concluded that the quality of life of the family was deeply related to the severity of AD, more than the quality of life of the children, pointing out to the deep impact of the condition on the family, in accordance with this study.^25^

There is a positive and moderate correlation (r=0.680) between the scores of CDLQI and the SCORAD index, as shown in a study conducted in the United Kingdom with 116 children, obtaining a significant correlation between the instruments in the two visits in which they were applied.^26^

The negative impact of AD on the lives of children, especially those with more severe conditions, calls the attention to the long-term effect caused by this disease, especially on the children’s behavior and development. So, these results bring out the possibility of using CDLQI as an extra measure to assess the disease in clinical practice,^26^ since the multidisciplinary approach, especially related to the quality of life of patients, increases the adherence of children and parents to the treatment, thus favoring a faster clinical evolution, reducing skin lesions.^27^

The observation of the correlation between the severity of the condition and the quality of life shown in this study was also confirmed in Montes Claros (MG), with children aged between 6 months and 5 years.^9^ Therefore, this study adds the information that the correlation between the severity of the disease and the quality of life is also effective in children in the age group of 5 to 16 years, living in the Amazon region, submitted to an environment marked by strong and frequent rains, high mean annual temperatures, and high air humidity. Besides, the socioeconomic factor is marked by low family income.

The authors recognize that this study had important limitations. Concerning the low sample power, the convenience sample was formed by 51 children of a target-population composed of 120 children. There was some restriction concerning the time frame for data collection (six months). The sample was taken from a single reference service, located in the metropolitan region of Belém (PA), lacking representativeness from other places in the Amazon region. Besides, the scale reading was required for some children who were not literate, and, in that case, the interviewer’s intermediation may have interfered in the understanding of the questions from the children approached in this study.

It is believed that more studies on the quality of life of patients with AD would be beneficial to establish multidisciplinary approaches (such as the indication of psychological follow-up when needed), aiming at elaborating strategies of treatment and promoting better control of the disease.
